# Decline in Titers of Anti-Idiotypic Antibodies Specific to Autoantibodies to GAD65 (GAD65Ab) Precedes Development of GAD65Ab and Type 1 Diabetes

**DOI:** 10.1371/journal.pone.0065173

**Published:** 2013-06-13

**Authors:** Helena Elding Larsson, Ida Jönsson, Åke Lernmark, Sten Ivarsson, Jared R. Radtke, Christiane S. Hampe, The Diabetes Prediction in Skåne (DiPiS), Type 1 Diabetes Trial Network

**Affiliations:** 1 Department of Clinical Sciences, Lund University, Malmö, Sweden; 2 Department of Pediatrics, University Hospital MAS, Malmö, Sweden; 3 Department of Medicine, University of Washington, Seattle, Washington, United States of America; La Jolla Institute for Allergy and Immunology, United States of America

## Abstract

The humoral Idiotypic Network consisting of antibodies and their anti-idiotypic antibodies (anti-Id) can be temporarily upset by antigen exposure. In the healthy immune response the original equilibrium is eventually restored through counter-regulatory mechanisms. In certain autoimmune diseases however, autoantibody levels exceed those of their respective anti-Id, indicating a permanent disturbance in the respective humoral Idiotypic Network. We investigated anti-Id directed to a major Type 1 diabetes (T1D)-associated autoantibody (GAD65Ab) in two independent cohorts during progression to disease. Samples taken from participants of the Natural History Study showed significantly lower anti-Id levels in individuals that later progressed to T1D compared to non-progressors (anti-Id antibody index of 0.06 vs. 0.08, respectively, p = 0.02). We also observed a significant inverse correlation between anti-Id levels and age at sampling, but only in progressors (p = 0.014). Finally, anti-Id levels in progressors showed a significant decline during progression as compared to longitudinal anti-Id levels in non-progressors (median rate of change: −0.0004 vs. +0.0004, respectively, p = 0.003), suggesting a loss of anti-Id during progression. Our analysis of the Diabetes Prediction in Skåne cohort showed that early in life (age 2) individuals at risk have anti-Id levels indistinguishable from those in healthy controls, indicating that low anti-Id levels are not an innate characteristic of the immune response in individuals at risk. Notably, anti-Id levels declined significantly in individuals that later developed GAD65Ab suggesting that the decline in anti-Id levels precedes the emergence of GAD65Ab (median rate of change: −0.005) compared to matched controls (median rate of change: +0.001) (p = 0.0016). We conclude that while anti-Id are present early in life, their levels decrease prior to the appearance of GAD65Ab and to the development of T1D.

## Introduction

The Network Hypothesis stated by Niels Jerne postulates that a network of interacting idiotypes is part of the immune regulatory system [1]. An antibody, which recognizes another antibody as its antigen is called an “anti-idiotypic antibody (anti-Id)”. A specific subclass of these anti-Id recognize the binding site of the original antibody and can thereby prevent the binding of the original antibody to its antigen. These “internal image antibodies” have been described in the control populations of several autoimmune diseases [2], [3] and may have regulatory functions. Importantly, anti-Id levels often correlate inversely with autoimmune disease activity. Thus high anti-Id levels are found during remission in patients with autoimmune diseases and low anti-Id levels are found during the acute, active phases of disease [4]–[6]. The development of anti-Id has been investigated in both animals and humans: an increase in antibody levels usually triggers a later increase in anti-Id level (reviewed in [7]). For example, Geha et al. showed that vaccination with tetanus toxoid not only induced the expected increase in antibodies against tetanus toxoid, but also triggered a later increase in anti-Id which tended to normalize the level of free tetanus toxoid antibody. This decrease of free tetanus toxoid antibody titer was caused by competition of anti-Id for the tetanus toxoid binding site on the idiotypic tetanus toxoid antibody [8], [9]. In addition, the development of anti-Id towards human antibodies that are used for therapy is a well documented phenomenon and a major concern for the biological availability of the therapeutic antibody [10]. While these studies show that anti-Id can be induced by administration of an exogenous antibody, or develop in response to an induced antibody increase, very little is known about the natural development of anti-Id.

Type 1 diabetes (T1D) is an autoimmune disease characterized by lack of insulin due to the autoimmune-mediated destruction of the islet beta cells. Progression to T1D is characterized by the appearance of circulating autoantibodies directed against several beta cell autoantigens: insulin, the smaller isoform of glutamate decarboxylase (GAD65), Insulinoma 2-associated protein, and the Zinc Transporter 8 protein (for review see [11]).

Previously we reported the concurrent presence of anti-Id specific to GAD65Ab and GAD65Ab in sera of healthy individuals [12]. Importantly, levels of anti-Id specific to GAD65Ab were significantly lower in T1D patients [12]. Finally, anti-Id levels increased in T1D patients who experienced the temporary remission known as the honeymoon phase. In contrast, patients who did not undergo remission did not show an increase of anti-Id levels [13]. Thus although there is an inverse correlation between beta cell specific autoimmunity and anti-Id levels after T1D has developed, it was not known if anti-Id levels actually declined before the development of T1D and whether this potential decline precedes the emergence of GAD65Ab.

To address these questions we investigated anti-Id in two longitudinal cohorts of individuals who progressed to T1D.

## Materials and Methods

### Ethics Statement

The Natural History Study (NHS): The protocol was approved by institutional review boards at participating centers (Benaroya Research Institute (Seattle), Children's Hospital Los Angeles, Columbia University, The George Washington University, Indiana University, Institut Fuer Diabetes (Munich), Joslin Diabetes Center, San Raffaele University (Milan), Stanford University, University of California San Francisco, University of Colorado, University of Florida, University of Miami, University of Minnesota, University of Pittsburgh, University of Texas Southwestern Medical School, University of Toronto, and the University of Washington), and all participants provided written informed consent before participation in the screening phase.

Diabetes Prediction in Skåne (DiPiS): The Regional Ethics Board in Lund, Sweden approved the DiPiS study. Written informed consent was obtained from participating individuals or their guardians.

### Patient cohorts

a) The Natural History Study (NHS) is part of the TrialNet initiative [14]. In this ongoing prospective study samples are collected from relatives of T1D probands. The age of the participants at enrollment ranges from 1–45 years. Participants are monitored annually or semi-annually, depending on their islet autoantibody status.

We analyzed longitudinal samples of 78 participants who developed T1D during the follow-up period (progressors) and 161 participants who remained disease-free throughout the analyzed time period (non-progressors) (Table 1).

**Table 1:** Sample information of the NHS cohort.

	Progressors	Non-progressors	P-value
N	78	161	
Age at initial sampling (years) (median and range)	12 (3–46)	12 (3–46)	NS
Duration of follow up (months) (median and range)	15.5 (2–60)	19 (5–61)	<0.0001
Number of available longitudinal samples (median and range)	3 (2–4)	4 (2–4)	<0.0001
Excluded subjects (n)	15	19	

b) Diabetes Prediction in Skåne (DiPiS) is a population-based prospective study of T1D in children born in the Swedish county of Skåne [15]. 46,000 children born between 2000 and 2004 were screened. Of these, 35,658 were HLA-typed resulting in the identification of more than 2,500 children with increased genetic risk for T1D, who were then followed annually, beginning at 2 years of age.

We analyzed longitudinal samples of 28 participants who progressed to T1D during the follow up period (progressors). Each progressor was matched for gender, HLA type, and birthdate with two individuals who remained diabetes-free during the follow-up period (non-progressors). We also analyzed 24 individuals who developed GAD65Ab during follow up, but remained diabetes-free (GAD65Ab converters). Each of these individuals was matched with two individuals who remained both diabetes-free and GAD65Ab-negative during the follow up period (Table 2). The follow up periods for progressors and GAD65Ab converters were carefully matched to those of their respective controls.

**Table 2:** Sample information of the DiPiS cohort.

	Progressors	Matched controls	GAD65Ab converters	Matched controls
N	28	56	24	48
Age at initial sampling (years) (median and range)	2	2 (2–4)	2 (2–4)	2 (2–4)
Duration of follow up (months) (median and range)	30 (6–90)	36 (12–84)	69 (35–133)	60 (36–84)

Data pertaining to this study are available upon request.

### Sample analysis

#### GAD65Ab

GAD65Ab were measured in a radioligand binding assay (RBA) [16], [17], as standardized in the International Combined Autoantibody Workshop [18]. Recombinant ^35^S-GAD65 was produced by *in vitro* coupled transcription/translation with SP6 RNA polymerase and nuclease-treated rabbit reticulocyte lysate (Promega, Madison, WI, USA) as previously described [16], [17]. The *in vitro* translated ^35^S-labeled antigen was kept at −70°C and used within 2 weeks. The serum samples were incubated overnight at 4°C in duplicate with TCA precipitable counts corresponding to 20,000 cpm of ^35^S-GAD65 at a final serum dilution of 1:25. Labeled antibody-bound antigen was separated from free antigen with Protein A-Sepharose (Zymed, San Francisco, CA, USA). Bound radioactivity was counted in a 1450 Microbeta (Wallac Oy, Turku, Finland) scintillation counter. The assay we used showed 70% sensitivity and 98% specificity for GAD65Ab, in the International Combined Autoantibody Workshop [18].

Antibody positive and negative samples were included in every assay to correct for inter-assay variation by calculating an autoantibody index (Ab-index  =  (cpm of tested sample – cpm of negative standards)/(cpm of positive standard – cpm of negative standards)) as described previously [16].

The upper limit (index of 0.05) of the normal range was established as described previously [19]. Briefly, we determined the lowest GAD65Ab index at which GAD65 binding by serum was specifically displaced by recombinant human GAD65 (Diamyd Medical AB, Sweden). In a cohort of 302 healthy individuals we observed seven samples with indices above 0.05, which corresponds to a percentile of 97.7.

The World Health Organization (WHO) standard [20] was used as a positive control. Samples with a GAD65Ab index above 20 were excluded from the analysis. These samples had to be diluted >10 times, introducing a potential large variability of error.

### Anti-Id to GAD65Ab

The complexes of GAD65Ab and anti-Id in serum samples were dissociated as described earlier [12], [21]. Briefly, serum samples (50 µl) were incubated with GAD65-specific human monoclonal antibody b96.11 coupled to Protein A-Sepharose (PAS) (50 µl of 10% slurry) for 10 min at 55°C. After this incubation the reaction was next incubated at 37°C for 30 min and then finally for 10 min at room temperature. The bead volume was titrated for optimal assay conditions. The bound fraction was separated from the unbound serum by centrifugation at 14,000 rpm for 5 min. The supernatant was carefully removed and re-centrifuged under the same conditions to ensure the absence of b96.11-PAS. The resulting supernatant was analyzed for GAD65Ab by RBA. Each assay included the WHO standard [20] that was not absorbed to b96.11-PAS, and a blank control, which consisted of PBS that had been absorbed to b96.11-PAS. Interassay variation of the WHO standard was ∼10%. An anti-Id index was calculated as the observed increase in GAD65Ab levels after absorption of the anti-Id compared to the GAD65Ab level before absorption of the anti-Id as previously reported [22]. Antibody levels were expressed as a relative index to correct for interassay variation. A positive control serum sample for anti-Id was run on every plate. This sample was obtained from a healthy donor. The median anti-Id index for this sample was 0.27 (range: 0.24–0.31) with a coefficient of variation of 6.83%.

The blank control verified that b96.11 was not released from the beads during the procedure. All samples obtained from one individual were analyzed on the same microtiter plate to avoid interassay variation.

#### Statistical analysis

All figures and statistical analyses were performed using Prism 4 (GraphPad Software, La Jolla, CA). Correlations were calculated using the Spearman rank correlation test. Median antibody levels between groups were compared using the non-parametric analysis of variance (Mann-Whitney-U test). Median antibody levels within groups were compared using the non-parametric analysis of variance (Wilcoxon-matched pair test). A p value of less than 0.05 was considered significant. Rate of change in antibody titer over time was calculated as follows: change in antibody titer (index)/time (months).

## Results

### Anti-Id levels in the NHS cohort

**Figure 1:**
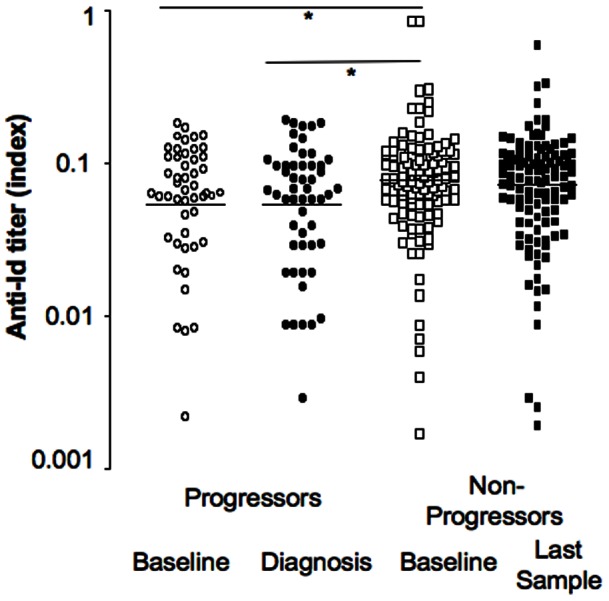
Anti-Id levels in progressors and non-progressors of the NHS cohort at baseline and at time of clinical diagnosis. Anti-Id levels and median for progressors at baseline (white circles) and at onset (black circles) and non-progressors at baseline (white squares) and at the last sample of the follow up (black squares) are presented. Significant differences between medians are indicated by horizontal bars and asterisks.

**Figure 2:**
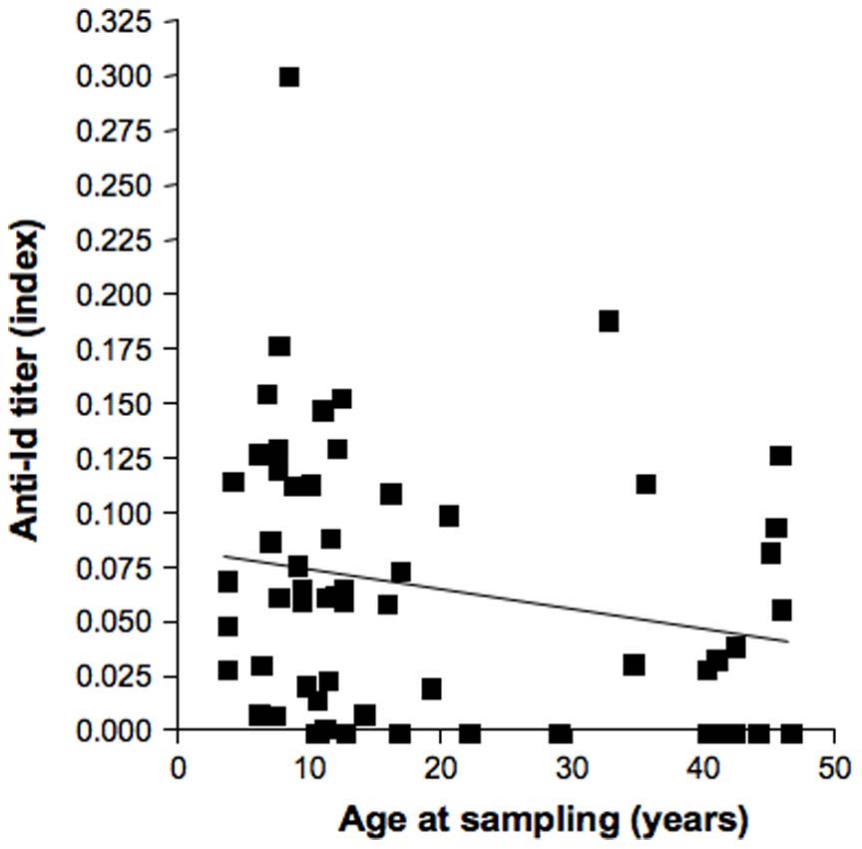
Anti-Id levels in progressors of the NHS cohort at baseline in relation to age at sampling. Anti-Id levels were plotted against age at sampling. Linear regression line is shown.

At baseline, patients in the NHS cohort who eventually progressed to diabetes (progressors) had significantly lower median anti-Id levels compared to non-progressors (index of 0.06 vs. 0.08, respectively, p = 0.02) (Figure 1). Also in the progressor group, anti-Id levels showed an inverse correlation with age at baseline in the progressor group (p = 0.014) (Figure 2). In contrast in the non-progressor group, there was no significant correlation between anti-Id level and age (data not shown).

**Figure 3:**
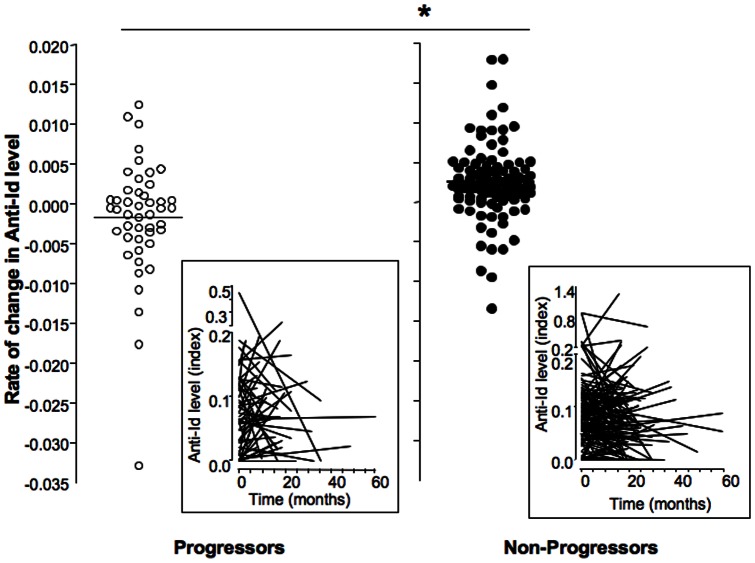
Longitudinal changes in anti-Id levels during follow up in progressors and non-progressors of the NHS cohort. Longitudinal changes are expressed as rate of change (anti-Id level at diagnosis or final sampling – anti-Id level at baseline)/duration of follow up in months). Changes in anti-Id levels and median for each group are presented. Significant differences are indicated by the horizontal bar and asterix. Actual anti-Id levels at baseline and over time are shown in the respective insets.

In the progressor group there was no significant difference between the anti-Id levels at baseline and those at diagnosis (median anti-Id level 0.061 vs. 0.060, respectively) (Figure 1), and anti-Id levels at baseline correlated significantly with those at diagnosis (p<0.0001). However, the follow-up periods ranged widely between subjects, (2 to 60 months), as did the anti-Id levels (anti-Id index  = 0–0.19) (Table 1). Therefore we calculated the rate of change of anti-Id level from baseline over time (delta anti-Id/month) (Figure 3).

We found that anti-Id levels fell over time in progressors but rose in non-progressors (median rate of change: −0.0004 vs +0.0004, respectively, p = 0.003).

Next we analyzed the changes in anti-Id levels in non-progressors who were at an increased risk of developing T1D as expressed by the presence of two or more autoantibodies. We found that significantly more individuals with increased risk showed decreasing anti-Id levels over time (15/45 = 33% vs. 17/90 = 19%, p = 0.02), while significantly more individuals with one or less autoantibodies showed stable anti-Id levels during the follow up period (43/90 = 48% vs. 11/45 = 24%, p<0.0001).

### GAD65Ab in the NHS cohort

**Figure 4:**
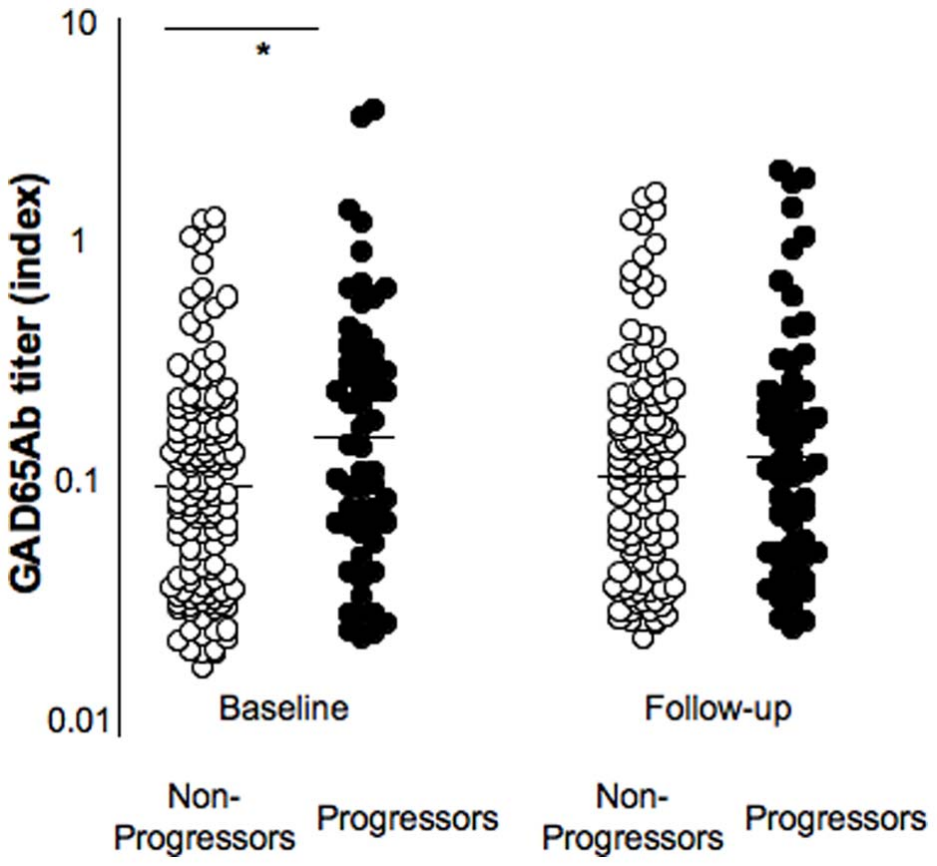
GAD65Ab level in progressors and non-progressors. GAD65Ab levels at baseline and at diagnosis (or final sampling in non-progressors) are shown for progressors and non-progressors of the NHS cohort. GAD65Ab levels and median for each group are presented. Significant differences are indicated by the horizontal bar and asterix.

The majority of both progressors and non-progressors were GAD65Ab-positive and only 26% (36/135) of non-progressors and 15% (9/59) of progressors were GAD65Ab-negative at baseline. Seventy percent (25/36) of GAD65Ab-negative non-progressors and 78% of GAD65Ab-negative progressors remained GAD65Ab-negative and 3% (4/135) and 5% (3/59) of non-progressors and progressors lost their GAD65Ab during the follow-up (Figure 4). GAD65Ab titers at baseline showed significant correlation with GAD65Ab titers at the end of the follow up period, both in progressors and non-progressors (p<0.0001). GAD65Ab levels at baseline were significantly higher in progressors compared to non-progressors (median index 0.18 vs. 0.12, respectively, p = 0.02), however no difference in GAD65Ab levels between the groups was observed at the end of the follow up period (median index at diagnosis and corresponding follow up sample in non-progressors: 0.15 and 0.12, respectively) (Figure 4).

### Anti-Id in DiPiS

For the analysis of the DiPiS cohort we were able to match two individual control subjects to each progressor or GAD65Ab-converter. Controls were matched for time until diagnosis, sex, and birthdate.

**Figure 5:**
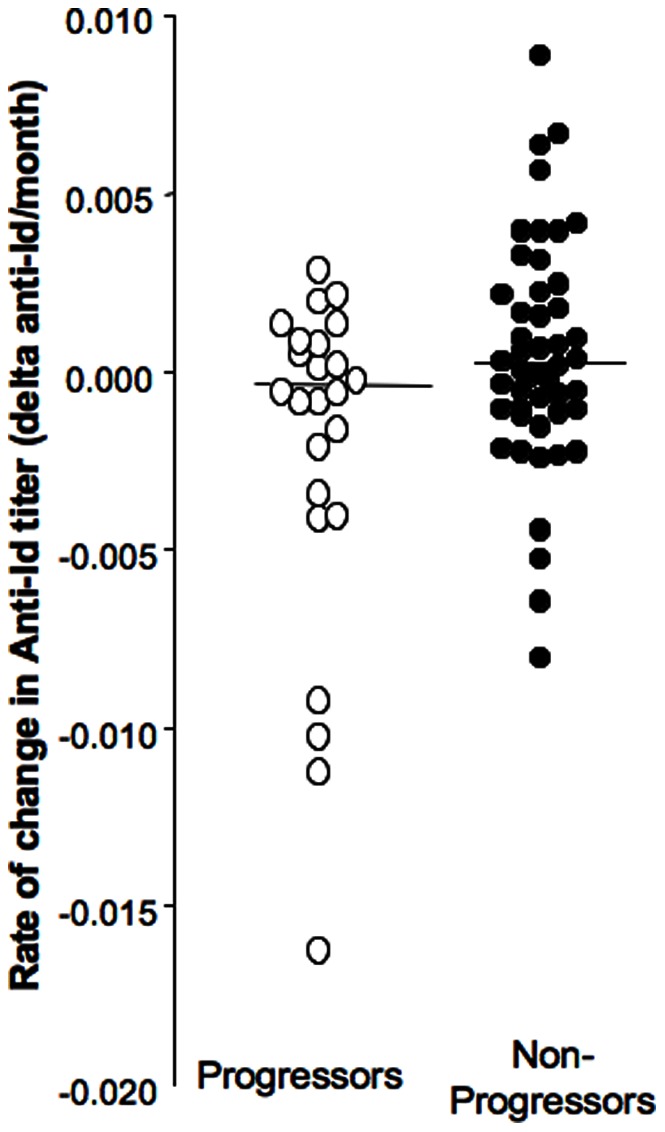
Longitudinal changes in anti-Id level during follow up in DiPiS progressors and matched non-progressors. Longitudinal changes in anti-Id levels over time are presented as rate of change (as described above). Changes in anti-Id level and median are shown for progressors (white circles) and matched non-progressors (black circles).

**Figure 6:**
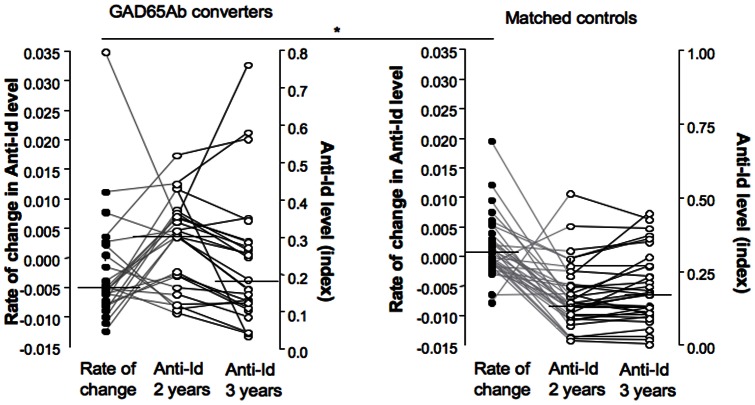
Longitudinal changes in anti-Id levels during the first measured interval (2–3 years of age) in GAD65Ab converters and matched controls. Changes in anti-Id over time are presented as rate of change (as described above) and are shown as black circles. For comparison to individual samples, anti-Id levels at ages 2 and 3 are shown as white circles. Significant differences in the medians are indicated by the horizontal bar and asterix.

As observed for the NHS cohort anti-Id levels at baseline correlated significantly with those at diagnosis (p<0.0001). In progressors in the DiPiS cohort, we found that anti-Id levels tended to decline over time (median rate of change: −0.0003), in contrast to matched non-progressors whose anti-Id levels tended to increase with time (median rate of change: +0.00025), similar to our finding in the NHS cohort. However, in the DiPiS cohort this difference in rate of change of anti-Id levels did not reach significance (p = 0.09) (Figure 5). Of special interest were 24 individuals who converted to GAD65Ab positivity but did not develop diabetes during the follow up period. In this group, there was no difference in anti-Id levels at baseline between GAD65Ab converters and their matched controls. However, during the first time interval studied (ages 2–3), GAD65Ab converters had a significant decrease in anti-Id levels (median rate of change: −0.005) in contrast to their matched controls who did not decrease (median rate of change: +0.001) (p = 0.0016) (Figure 6). Of note, this decline in anti-Id occurred prior to the appearance of GAD65Ab.

## Discussion

We investigated anti-Id reactive to the T1D-specific GAD65Ab b96.11 in two longitudinal cohorts of individuals that progressed to T1D. Samples taken at baseline from participants of the NHS cohort showed significantly lower anti-Id levels in those individuals that later progressed to T1D compared to non-progressors. We also found an inverse correlation between age and anti-Id levels in the progressors, in contrast to non-progressors. Thus there appears to be an age-dependent decline of anti-Id in individuals at risk of developing T1D. Furthermore our longitudinal analysis of progressors and non-progressors in this cohort showed a significant decline in anti-Id levels with time in progressors in contrast to non-progressors.

Our analysis of the DiPiS cohort samples showed that early in life (age 2) individuals that later progressed to T1D have anti-Id levels indistinguishable from those in controls that remained healthy. This indicates that low anti-Id levels are not an innate characteristic of the immune response in individuals at risk. However thereafter anti-Id levels declined significantly with time in individuals that later developed GAD65Ab. From these data we conclude that while anti-Id are present early in life, their levels decrease prior to both the emergence of GAD65Ab and the development of T1D.

While the increase in antibody titer induced by a temporary exposure to antigen triggers a subsequent increase of anti-Id and thereby restores the equilibrium, prolonged release of GAD65, e.g. during destruction of beta cells, will continuously activate polyclonal GAD65-specific B cells and induce the secretion of GAD65Ab. Somatic mutations and affinity maturation will change the secreted GAD65Ab idiotypes and eventually the anti-Id response will fail to respond adequately to these changes. This hypothesis is supported by a recent publication, where the idiotypic network failed after hyperimmunization of mice with tetanus toxoid [23].

We acknowledge that our study has several limitations. First, even the non-progressors in both cohorts are at an increased risk of developing T1D. For example, since the NHS non-progressors are relatives of T1D patients they have an average risk of ∼6% compared to <1% in the general population [24], [25]. Likewise the DiPiS non-progressors are genetically susceptible individuals with an overall risk of 6% [26], [27]. Indeed we observed that anti-Id levels in non-progressors of the NHS cohort, who had an increased risk for the development of T1D as expressed by the presence of two or more autoantibodies, more often decreased over time as compared to non-progressors, who presented with one or less autoantibody. Since a sizable portion of the non-progressors in both studies will eventually progress to T1D, it is remarkable that we were still able to detect the reported differences between progressors and non-progressors.

Another limitation of the NHS cohort was that the majority of non-progressors were GAD65Ab-positive, indicating the presence of an ongoing GAD65-specific autoimmunity. Our analysis of the individuals in the DiPiS cohort that converted to GAD65Ab positivity during the follow-up suggests that anti-Id levels decline early in life prior to development of GAD65Ab. Therefore the presence of GAD65Ab in the non-progressors of the NHS cohort suggests that a major decline in anti-Id levels had already occurred in these individuals by the time we sampled them and a steeper decline of anti-Id levels may be observed in longitudinal analyses of progressors that are GAD65Ab-negative at baseline. It is unfortunate that neither cohort contained a sizable portion of progressors who converted to GAD65Ab-positivity prior to development of T1D. The 24 healthy GAD65Ab converters of the DiPiS cohort will be monitored for the development of T1D as these individuals are at an increased risk.

The strengths of these cohorts lay primarily in the careful collection of well characterized longitudinal samples of progressors and the availability of controls that could be matched not only for sex and HLA (NHS cohort), but also for birthdate and the period of follow up (DiPiS cohort).

We conclude that anti-Id to GAD65Ab are lost during progression to T1D and prior to the development of other markers of the autoimmune process, such as GAD65Ab. Despite these intriguing observations, the extent to which anti-Id can be used in the prediction of T1D remains to be investigated, as does their potential function as an immune regulator.

## Supporting Information

Supplement S1 **Members of the DiPiS study group and the Type 1 Diabetes Trial Network.**
(DOC)
