# Supplementary material for: Decline in Titers of Anti-Idiotypic Antibodies Specific to Autoantibodies to GAD65 (GAD65Ab) Precedes Development of GAD65Ab and Type 1 Diabetes
Source: PLoS One. 2013 Jun 13;8(6):e65173. doi: 10.1371/journal.pone.0065173 (PMC3681789; doi:10.1371/journal.pone.0065173)
Supplement: Supplement S1 — Members of the DiPiS study group and the Type 1 Diabetes Trial Network. (DOC) [file pone.0065173.s001.doc]

Members of the DiPiS Study Group:

Cecilia Andersson (Malmö), Charlotte Brundin (Malmö), Annelie Carlsson (Lund), Elisabeth Cederwall (Ängelholm), Corrado Cilio (Malmö), Helena Elding Larsson (Malmö), Gertie Hansson (Malmö), Sten Ivarsson (Malmö), Berglind Jonsdottir (Malmö), Björn Jönsson (Ystad), Ida Jönsson (Malmö), Karin Larsson (Kristianstad), Barbro Lernmark (Malmö), Åke Lernmark (Malmö), Bengt Lindberg (Malmö), Zeliha Mestan (Malmö), Jan Neiderud (Helsingborg), Anita Nilsson (Malmö), Tore Vigard, (Ystad), Ingrid Wigheden (Malmö).

Members of the Type 1 Diabetes TrialNet Study Group

Steering Committee: Jay S. Skyler (University of Miami Diabetes Research Institute), Chairman; Mark Anderson (University of California San Francisco), Katarzyna Bourcier (NIAID), Dorothy Becker (University of Pittsburgh), Penelope Bingley (University of Bristol), Janice Blum (Indiana University), Emanuele Bosi (San Raffaele Hospital), Jane Buckner (Benaroya Research Institute), H. Peter Chase (University of Colorado Barbara Davis Center for Childhood Diabetes), Michael Clare-Salzler (University of Florida), Peter Colman (Walter and Eliza Hall Institute of Medical Research), Linda DiMeglio (Indiana University), George S. Eisenbarth (University of Colorado Barbara Davis Center for Childhood Diabetes), C. Garrison Fathman (Stanford University), Stephen Gitelman (University of California San Francisco), Robin Goland (Columbia University), Peter Gottlieb (University of Colorado Barbara Davis Center for Childhood Diabetes), Gilman Grave (NICHD), Carla Greenbaum (Benaroya Research Institute), Leonard Harrison (Walter and Eliza Hall Institute of Medical Research), Kevan Herold (Yale University), Richard Insel (Juvenile Diabetes Research Foundation), Jeffrey P. Krischer (University of South Florida), Jeffrey Mahon (University of Western Ontario), Jennifer Marks (University of Miami Diabetes Research Institute), Antoinette Moran (University of Minnesota), Jerry P. Palmer (University of Washington), Mark Peakman (Guy’s, King’s, and St. Thomas’ School of Medicine), Alberto Pugliese (University of Miami Diabetes Research Institute), Philip Raskin (University of Texas Southwestern Medical School), Maria Grazia Roncarolo (San Raffaele Scientific Institute), William Russell (Vanderbilt University), Peter Savage (NIDDK), Desmond Schatz (University of Florida), Robert Sherwin (Yale University), Mark Siegelman (University of Texas Southwestern Medical School), Olli Simell (Hospital District of Southwest Finland), James Thomas (Vanderbilt University), Massimo Trucco (University of Pittsburgh), John Wagner (University of Minnesota), Diane Wherrett (University of Toronto), Darrell M. Wilson (Stanford University), William Winter (University of Florida), Judith Fradkin (NIDDK, ex-officio), Ellen Leschek (NIDDK, ex-officio), Lisa Spain (NIDDK, ex-officio).

Past Members: Christophe Benoist (Joslin Diabetes Center), Jeffrey Bluestone (University of California San Francisco), David Brown (University of Minnesota), Catherine Cowie (NIDDK), Bernard Hering (University of Minnesota), Stanley Jordan (Cedars-Sinai Medical Center), Francine R. Kaufman (Childrens Hospital Los Angeles), John M. Lachin (George Washington University), Kirsti Nanto-Salonen (Hospital District of Southwest Finland), Gerald Nepom (Benaroya Research Institute), Tihamer Orban (Joslin Diabetes Center), Robertson Parkman (Childrens Hospital Los Angeles), Mark Pescovitz† (Indiana University), John Peyman (NIAID), John Ridge (NIAID), Henry Rodriguez (Indiana University), Anette Ziegler (Institut für Diabetesforschung).

Executive Committee: Jay S. Skyler, Katarzyna Bourcier, Carla J. Greenbaum, Jeffrey P. Krischer, Ellen Leschek, Lisa Rafkin (University of Miami Diabetes Research Institute), Peter Savage, Lisa Spain.

Past Members: Catherine Cowie, Mary Foulkes (George Washington University), Heidi Krause- Steinrauf (George Washington University), John M. Lachin, Saul Malozowski (NIDDK), John Peyman, John Ridge, Stephanie J. Zafonte (George Washington University).

Chairman's Office: Jay S. Skyler, Carla J. Greenbaum, Norma S. Kenyon, Lisa Rafkin, Irene Santiago, Jay M. Sosenko

TrialNet Coordinating Center (University of South Florida): Jeffrey P. Krischer, Brian Bundy, AQesha Luvon Ritzie, Michael Abbondondolo, Timothy Adams, Persida Alies, Franz Badias, Craig Beam, Matthew Boonstra, David Boulware, David Cuthbertson, Christopher Eberhard, Julie Ford, Jinin Ginem, Heather Guillette, Brian Hays, Martha Henry, Pat Law, Cristin Linton, Shu Liu, Jennifer Lloyd, Sarah Muller, Ryan O’Donnell, Yazandra Parrimon, Kate Paulus, Jennifer Pilger, Joy Ramiro, Amy Roberts, Kelly Sadler, Amanda Terry, Margaret Wootten, Ping Xu, Kenneth Young

Past Staff Members: Monica Bassi, Doug Freeman, Moriah Granger, Michelle Kieffer, Lavanya Nallamshetty, Audrey Shor

Previous Coordinating Center (George Washington University) (who were involved with study at time of initiation): John M. Lachin, Mary Foulkes, Pamela Harding, Heidi Krause-Steinrauf, Susan McDonough, Paula F. McGee, Kimberly Owens Hess, Donna Phoebus, Scott Quinlan, Erica Raiden

NIDDK Staff: Judith Fradkin, Ellen Leschek, Peter Savage, Lisa Spain

Data Safety and Monitoring Board: Emily Blumberg (University of Pennsylvania), Chair; Jonathan Braun (University of California Los Angeles), Lori Laffel (Joslin Diabetes Center), Ali Naji (University of Pennsylvania), Jorn Nerup (University of Copenhagen), Trevor Orchard (University of Pittsburgh), Anastasios Tsiatis (North Carolina State University), Robert Veatch (Georgetown University), Dennis Wallace (Research Triangle Institute).

Past Members: Ake Lernmark (Lund University), Bernard Lo (University of California San Francisco), Herman Mitchell (Rho Inc.), Michael Steffes (University of Minnesota), Bernard Zinman (University of Toronto).

Infectious Disease Safety Committee: Brett Loechelt (Children's National Medical Center) (Medical Monitor), Lindsey Baden (Harvard University), Michael Green (University of Pittsburgh), Adriana Weinberg (University of Colorado)

Laboratory Directors: George S. Eisenbarth, Santica Marcovina (University of Washington), Jerry P. Palmer, Adriana Weinberg, William Winter, Liping Yu (University of Colorado Barbara Davis Center for Childhood Diabetes), Sunanda Babu (University of Colorado Barbara Davis Center for Childhood Diabetes)

Protocol Advisory Committee: Tihamer Orban (Chair), Peter Gottlieb, Carla Greenbaum, Heidi Krause-Steinrauf, John M. Lachin, Ellen Leschek, Brett Loechelt, Robertson Parkman, Lisa Rafkin, Alison Rigby, Jay S. Skyler, Lisa Spain, John Wagner.

North American Clinical Center Staff involved in this Protocol:

Benaroya Research Institute, Seattle, Washington: Carla Greenbaum, Jennifer Bollyky, Srinath Sanda, David Tridgell, Marli McCulloch-Olson, Heather Vendettuoli, Deborah Hefty, Mary Ramey, Christine Webber, Kristen Kuhns, Nicole Hilderman, Angela Dove, Marissa Hammond, Jani Klein, Emily Batts

Childrens Hospital Los Angeles: Roshanak Monzavi, Mary Halvorson, Meredith Bock, Lynda Fisher, Debra Jeandron, Jamie Wood, Francine R. Kaufman

Columbia University, New York: Robin Goland, Ellen Greenberg, Mary Pat Gallagher, Jeniece Trast, Mary Chan

Indiana University, Indianapolis: Henry Rodriguez, Mark Pescovitz, Linda DiMeglio, Lyla Christner, Maria Nicholson, Martha Mendez

Joslin Diabetes Center, Boston: Tihamer Orban, Christophe Benoist, Joseph Wolfsdorf, Alyne Ricker, Heyam Jalahej, Debbie Conboy, Klara Farkas, Janos Kis, Hui Zhang, Steve Fay

Stanford University, California: Darrell M. Wilson, Bruce A. Buckingham, Tandy Aye, Trudy Esrey, Adriana Soto, Jennifer Perry, Bonita Baker, Alison Rigby, Barbara Berry

University of California San Francisco: Stephen E. Gitelman, Stephen M. Rosenthal, Mark Anderson, Saleh Adi, Kathleen Breen, Celia Hamilton

University of Colorado Barbara Davis Center for Childhood Diabetes, Aurora, Colorado: Peter Gottlieb, H. Peter Chase, Aaron Michels, Whitney Kastelic, Laurie Weiner

University of Florida, Gainesville, FL: Desmond Schatz, Michael Haller, Michael Clare-Salzler, Roberta Cook, Diane Mancini, Annie Abraham, Elena Hicks, Gloria Cole

University of Miami Diabetes Research Institute, Miami, Florida: Jennifer B. Marks, Alberto Pugliese, Della Matheson, Carlos Blaschke, Luz Arazo, Mario Cisneros, Brenda Acosta

University of Minnesota, Minneapolis: Antoinette Moran, Brandon Nathan, John Wagner, Mary Ann Boes, Carrie Gibson, Lois Finney, Theresa Albright-Fischer, Jennifer Smith

University of Pittsburgh, Pennsylvania: Dorothy Becker, Frederico Toledo, Ingrid Libman, Karen Riley, Kelli Delallo, Kym Smith, Diane Gwynn, Gyna Wohlers

University of Texas Southwestern Medical School: Philip Raskin, Perrin White, Bryan Dickson, Soumya Adhikari, Mark Siegelman, Marilyn Alford, Nenita Torres, Tauri Harden, Lourdes Pruneda, Erica Cordova, Renee Davis, Stefani Fernandez, Jamie Arthur

University of Toronto: Diane Wherrett, Lesley A. Eisel, Brenda Ahenkorah, Natasha Razack, Mithula Sriskandarajah

Vanderbilt University: William E. Russell, James W. Thomas, Daniel J. Moore, Anne Brown, Margo Black, Eric Pittel, Faith Brendle

International Clinical Center Staff involved in this Protocol:

San Raffaele Hospital (Italy): Emanuele Bosi, Manuela Battaglia, Cristina Belloni, Eleonora Bianconi, Luca Falqui, Pauline Grogan, Carlo Lombardoni, Sabina Martinenghi Pauline Grogan

University of Bristol (United Kingdom): Penelope Bingley, Rachel Aitken, Harriet Castleden, Nicola Farthing, Claire Matthews, Jenny McGee, Carole Wheaton

Walter and Eliza Hall Institute of Medical Research (Australia): Leonard C. Harrison, Elena Andaloro, Candice Breen, Peter Colman, Spiros Fourlanos, Shane Gellert, Felicity Healy, John Wentworth

Hospital District of Southwest Finland (Finland): Oilli G. Simell, Sanna Jokipuu, Tiina Kallio, Elina Mantymaki, Kirsti Nanto-Salonen, Tiina Niininen, Birgitta Nurmi, Minna Romo, Eeva Ruohonen, Maria Sarma, Tuula Simell, Sointu Suomenrinne, Maija Torma
